# Supplementary material for: Content-rich biological network constructed by mining PubMed abstracts
Source: BMC Bioinformatics. 2004 Oct 8;5:147. doi: 10.1186/1471-2105-5-147 (PMC528731; doi:10.1186/1471-2105-5-147)
Supplement: Additional File 5 — The original Chilibot query results of the term "long-term potentiation (LTP)" and 22 other terms, limiting the latest references analyzed to the years 1990, 1995, 2000, and 2004. [file 1471-2105-5-147-S5.bz2 › chilibotAdditionalFile5/ltp2000/html/ERK_ATF.html]

 


 **ERK** and **ATF** 
  
Found 26 abstracts in PubMed,  **26 abstracts were retrieved and analyzed**.  


---

 Search Google  |
 PDF files only 
|  EDU domain only 

---

**Interactive relationship** (e.g. stimulation, inhibition, etc)

- IL 1 beta... caused phosphorylation of the substrates Elk 1,  **ATF**  2 and hsp25, and the phosphorylation of both Elk 1 and hsp25 were decreased by the p38 blocker SB203580 p38i and the MAPK  **ERK**  blocker PD 098059 MEKi.  Ref: 10903806 Eur Cytokine Netw, 2000

- :-)

  **Parallel relationship** (e.g. studied together, co-existance, homology, etc.)

  - On the basis of resistance to the growth factor receptor inhibitor suramin and of several types of cross refractoriness experiments, the UVC induced CREB  **ATF**  1 phosphorylation represents an as yet unrecognized route of UVC induced signal transduction, independent of suramin inhibitable growth factor receptors and different from the  **Erk**  p62TCF pathway.  Ref: 9118940 EMBO J, 1997
  - The analysis of two distinct mitogen activated protein kinase pathways shows that stress activated protein kinase Jun N terminal kinase activation, resulting in the phosphorylation of  **ATF**  2, c Jun, and JunD, is required not only for the IL 1 but also for the TPA dependent induction, while the extracellular signal related kinase 1  **ERK**  1 and  **ERK**  2 activation is involved in the TPA but not in the IL 1 dependent stimulation of the uPA enhancer.  Ref: 10454570 Mol Cell Biol, 1999
